# Spontaneous retroperitoneal hematoma: a rare bleeding occurrence in COVID-19

**DOI:** 10.1093/omcr/omab081

**Published:** 2021-09-13

**Authors:** Whei Chuern Yeoh, Kee Tat Lee, Nadiah Hanim Zainul, Sharifah Baizura Syed Alwi, Lee Lee Low

**Affiliations:** 1 Medical Department, Hospital Sultanah Bahiyah, Kedah, Malaysia; 2 Infectious Disease Unit, Medical Department, Hospital Sultanah Bahiyah, Kedah, Malaysia

## Abstract

Emerging evidence suggest that COVID-19 is associated with hypercoagulability, predisposing patients to increase risk of thromboembolism. Anticoagulation is not without its risks of bleeding and decision to initiate anticoagulation should be carefully considered with close monitoring. Spontaneous retroperitoneal hematoma is a rare complication, and there are only a few documented reports implicating anticoagulant or antiplatelet agents as a potential cause. We report a 57-year-old gentleman with COVID-19 pneumonia who developed hypotension on Day 10 of illness while on prophylactic anticoagulation. Computed tomography scan of abdomen revealed a large right retroperitoneal and psoas muscle hematoma and he underwent surgical exploration to evacuate the hematoma. His condition improved and was discharged well. Although prophylactic anticoagulation may reduce thrombotic complications in severely ill COVID-19 patients, a high index of suspicion for rare bleeding complications should be maintained if patients become hemodynamically unstable. Early diagnosis and appropriate intervention may improve outcome and prevent mortality.

## INTRODUCTION

COVID-19, caused by SARS-CoV-2 virus, has caused a global pandemic since late 2019. Since its outbreak, COVID-19 has shown many different clinical manifestations. Although thrombosis is one of the hallmarks of this disease, the real incidence of bleeding in COVID-19 remains unknown due to lack of data reported in the literature. Thromboembolic events have been shown to be as high as 21% with a mortality rate of ~74% in COVID-19 infected individuals [1]. Anticoagulants are frequently used in severe COVID-19 infection to prevent thrombosis and it has been shown to reduce mortality [2]. However, the dosage, timing and duration of anticoagulation as well as the drug of choice remains an area of much debate due to lack of definite guidelines to date. Moreover, the use of anticoagulation is not without its risk. Currently, there are several studies including REMAP-CAP trial, ATTACC and ACTIV-4 trial who are investigating the role of empirical anticoagulation in all patients with severe COVID-19 infection. The bleeding complications can range from minor to major or even life-threatening conditions.

We present an interesting case of large retroperitoneal and psoas hematoma while on prophylactic anticoagulation for severe COVID-19 infection.

## CASE REPORT

A 57-year-old gentleman with no known medical illness presented with history of fever, abdominal pain, diarrhea and non-productive cough for one week. Vital signs on admission revealed blood pressure of 147/83 mmHg, pulse rate of 81 beats per minute, temperature of 36.3°C and oxygen saturation of 96% under room air. His physical examination revealed that he is tachypnoeic with a respiratory rate of 26 breaths per minute with minimal coarse crackles over the right lower zone of the lung. Laboratory investigations showed that he has mild acute kidney injury on presentation associated with lymphopenia and elevated inflammatory markers (Table 1). His initial chest X-ray showed bilateral lung infiltrates. Nasopharyngeal and oropharyngeal swabs for reverse-transcription polymerase chain reaction were detected positive to severe acute respiratory syndrome coronavirus 2 (SARS-CoV2). He was promptly started on intravenous ceftriaxone, dexamethasone, prophylactic dose of subcutaneous enoxaparin (40 mg daily) and supplemental oxygen with nasal cannula.

**Table 1:** Laboratory results

	Admission	Day 8	Day 10	Day 15	Day 20	References
White blood cells	5.73	5.33	9.67	7.02	9.12	4–10 × 10^9^/L
Hemoglobin	11.1	12.9	5.2	14.7	15.6	13–17 g/dl
Platelet	208	152	163	68	184	150–410 × 10^9^/L
Absolute neutrophil	4.89	4.59	7.19	5.94	6.82	2–7 × 10 ^9^/L
Absolute lymphocyte	0.46	0.41	1.81	0.49	1.35	1–3 × 10 ^9^/L
Prothrombin time	16.6	-	19.5	16.4	-	11.6–14.6 sec
International normalization ratio	1.28	-	1.52	1.27	-	1–1.2
Activated partial thromboplastin time	45.9	-	42.3	47.8	-	32.3–46.2 sec
D-dimer	5.38	-	2.58	6.96	-	0–0.5 ug/ml
Fibrinogen	-	-	291	423	-	150–450 mg/dl
Urea	12.8	5.5	11.5	5.5	8.6	3.2–8.2 mmol/L
Sodium	129	139	140	144	139	132–146 mmol/L
Potassium	4.0	4.1	4.1	3.7	3.7	3.5–5.5 mmol/L
Creatinine	118	70	109	86	81	62–115 umol/L
CRP	119.6	21.42	20.61	>156	44.88	0–5 mg/L
Ferritin	581	-	1153	674	-	22–322 ng/ml
Interleukin 6	-	-	30.5	71.7	-	0–4.4 pg/ml

**Figure 1:**
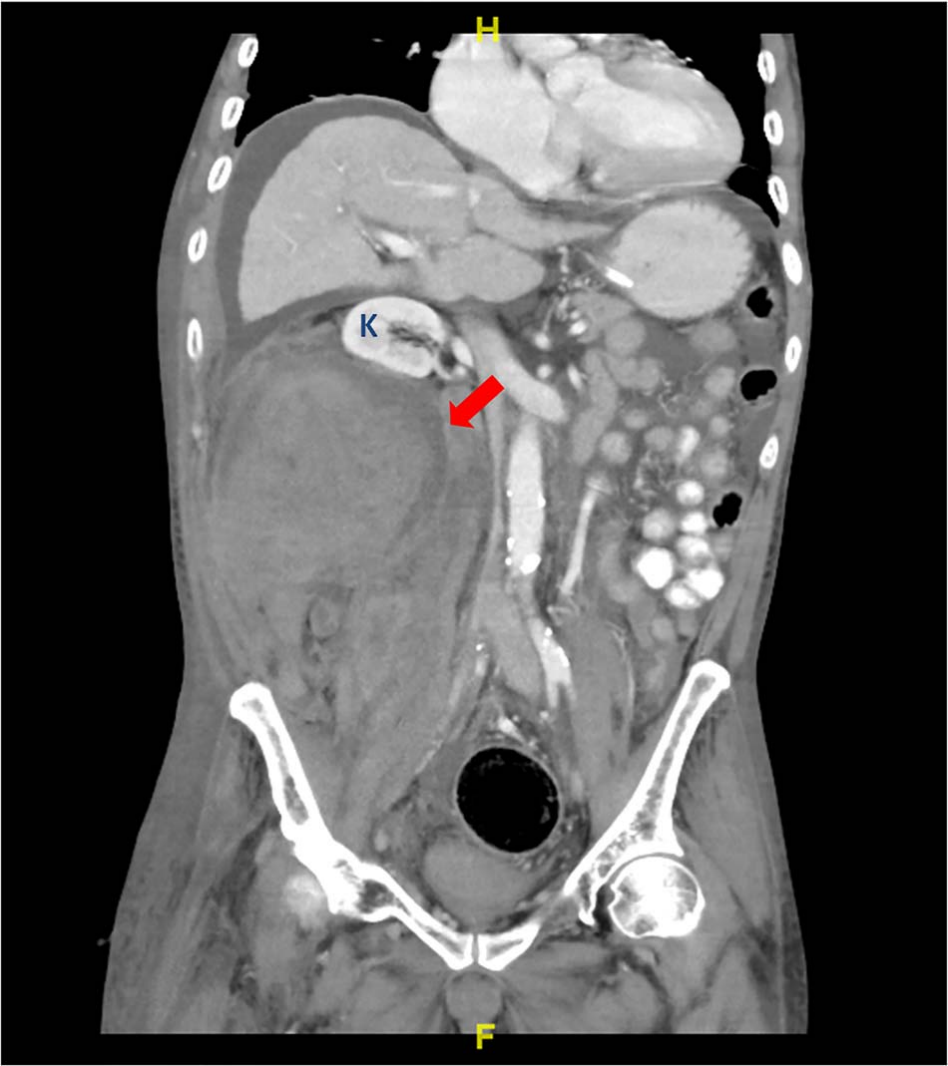
CT abdomen coronal view shows large right retroperitoneal hematoma (red arrow) involving right psoas muscle displaced right kidney (K) anterosuperiorly.

**Figure 2:**
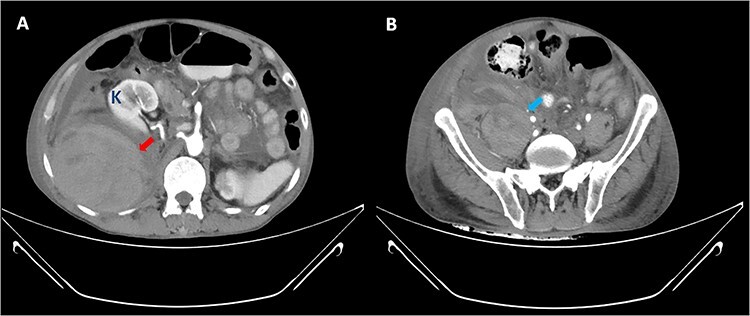
CT abdomen axial view shows large right retroperitoneal hematoma (red arrow) and right psoas hematoma (blue arrow) with displacement of right kidney (K) anteriorly.

**Table 2:** Summary of clinical characteristics, treatment and outcome of COVID-19 patients with retroperitoneal hematoma

Case	Age/Sex	Comorbidity	Clinical presentation	Site of bleeding	Anticoagulation	Medical therapy	Intervention	Outcome
1 [4]	88/male	Vascular dementia, atrial flutter, ischaemic heart disease (IHD)	Right lower abdominal pain, palpable right iliac fossa mass	Rectus sheath hematoma	Therapeutic enoxaparin	Reversed with protamine sulfate	No	Alive
2 [4]	85/female	Not mentioned	Respiratory failure Incidental finding during computed tomography of the pulmonary angiogram (CTPA)	Left sided retroperitoneal hematoma	Not mentioned	Blood transfusion and reversal of anticoagulation	No	Not mentioned
3 [4]	66/male	Obstructive sleep apnoea, pulmonary hypertension, chronic obstructive pulmonary disease (COPD), atrial fibrillation (AF), obesity	Hypotension and anemia	Left sided retroperitoneal hematoma	Therapeutic enoxaparin	Volume resuscitation, blood transfusion and reversal of anticoagulation	No	Died
4 [5]	69/male	diabetes mellitus (DM), hypertension (HPT), IHD	Abdominal pain, hypovolemic shock (Day 20 admission)	Right psoas hematoma	Prophylactic enoxaparin (40 mg OD) Therapeutic enoxaparin (1 mg/kg) due to worsening hypoxemia with elevated d-dimers	Volume resuscitation, packed red blood cell (PRBC) and fresh frozen plasma (FFP) transfusion	Arterial embolization	Alive
5 [6]	77/male	HPT, dyslipidemia, AF (on apixaban)	Hemodynamic instability and anemia (D14 admission)	Left retroperitoneal hematoma	Prophylactic enoxaparin (100 UI/kg OD)	Volume resuscitation, blood transfusion, continuous renal replacement therapy (CRRT)	Surgical evacuation of hematoma	Alive
6 [7]	62/male	Not mentioned	Left lower back pain (D14 admission)	Left iliopsoas hematoma + retroperitoneal hematoma	Recombinant human soluble thrombomodulin (rhsTM; 12 800 U twice daily) X 5/7 Then Enoxaparin 40 mg OD	PRBC transfusion	Arterial embolization	Alive
7 [7]	79/male	Not mentioned	Right lower back pain Hypovolemic shock	Right iliopsoas hematoma + retroperitoneal hematoma	Intravenous UFH	PRBC transfusion	Arterial embolization	Died
8 [8]	65/male	DM, HPT	Right flank pain	Large retroperitoneal hematoma	IV Heparin 5000 u/6 H	Volume resuscitation	No	Alive

Patient initially showed signs of improvement as he has improving cough and exertional dyspnoea with reduction in his C-reactive protein (CRP) levels. However, decision was made to continue him on prophylactic dose of enoxaparin as he was still dependent on nasal cannula for oxygen supplementation and his serial chest X-rays did not show remarkable improvement. Moreover, he was not ambulating well during his stay in ward. On Day 10 of illness, he developed hemodynamic instability with a sharp decrease in hemoglobin concentration from 12.9 to 5.2 g/dl. On clinical examination, he was tachypnoeic and pale with poor peripheral perfusion. Otherwise, other physical examination was unremarkable. There was no evidence of upper and lower gastrointestinal tract bleeding. Other laboratory investigations showed normal platelet count with severe lactic acidosis (serum lactate of 13.6 mmol/L, serum bicarbonate levels of 12 mmol/L). He was intubated for respiratory distress and commenced on blood transfusion. A contrast-enhanced computed tomography (CT) scan of his abdomen revealed a large right retroperitoneal and right psoas muscle hematoma (Figs 1 and 2) measuring ~12.5 cm anterior posterior (AP) × 12.4 cm (W) × 22.4 cm craniocaudal (CC) extending from kidney level at T12 until the right iliac fossa region, pushing the right kidney anterior and superiorly with evidence of active bleed within the hematoma. He underwent surgical exploration, the retroperitoneal hematoma was evacuated and hemostasis secured with abdominal packing. The patient required vasopressor support for the next 48 hours and was transfused with 14 units of packed cell, 6 units of fresh frozen plasma, 12 units of cryoprecipitate and 18 units of platelet in total. He underwent another revision surgery 2 days later and the abdominal packing was removed. His general condition and blood parameters improved remarkably thereafter and he was discharged well.

## DISCUSSION

The risk of developing thrombosis in COVID-19 has been postulated to be triggered by the attachment of SARS-CoV-2 virus to the angiotensin-2 receptor of the endothelial cells, leading to release of proinflammatory cytokines, endothelial dysfunction and systemic inflammation. Although administration of anticoagulation may prove beneficial in COVID-19 infection, it has been associated with bleeding events. A study by Al-Samkari *et al*. [3] has shown that the overall bleeding incidence rate in COVID-19 patients were 4.8 and 7.6% in non-critically ill and critically ill patients respectively, with major bleeding rates (WHO Grade 3–4) of 2.3%.

Recently, there are several papers reporting similar presentation of retroperitoneal hemorrhage as a complication of COVID-19. We identified five published case reports describing eight cases of bleeding events in COVID-19 [4–8]. Including our case, patients who developed retroperitoneal hematoma had a mean age of 73.9 years old, and seven patients (87.5%) were males. Out of the eight patients described, three patients were being treated with arterial embolization and one patient underwent surgical exploration, whereas the remaining patients were being treated conservatively. Five out of the eight patients survived and two patients died, whereas the outcome of the one patient was not mentioned (Table 2).

The diagnosis of retroperitoneal hematoma requires a high degree of clinical suspicion as patients do not exhibit any clinically apparent signs and symptoms until a substantial amount of blood loss has occurred. It should be suspected in patients who present with significant groin, flank, abdominal, back pain or hemodynamic instability after an interventional procedure or in patients who are anticoagulated. A contrast-enhanced CT scan of the abdomen remains the imaging modality of choice in the diagnosis of retroperitoneal hemorrhage as it serves to identify the anatomical extension, size of hematoma and compressive complications or extravasation. The mainstay management of retroperitoneal hematoma consists of cessation or modification of anticoagulation therapy and volume resuscitation with fluid and blood products. Small hematomas with mild symptoms without displacement of retroperitoneal structures and without need for multiple blood transfusions may be treated conservatively. Other treatment options such as selective arterial embolization via interventional radiology or surgical exploration with evacuation of hematoma are reserved for patients with significant abdominal hypertension symptoms and who are hemodynamically unstable [9].

There is no clear consensus regarding the management of retroperitoneal hematoma. Early detection and prompt intervention would result in a better outcome. We postulate that early intervention will reduce the expansion of hematoma and shorten the duration of compression of the surrounding tissues. There are controversies regarding prophylactic anticoagulation in patients with COVID-19 pneumonia. However, British Thoracic Society and Scottish Intercollegiate Guidelines Network suggest the use of prophylactic dose low molecular-weight heparin (LMWH) for patients who require admission and intermediate-dose LMWH (twice daily standard prophylactic dose) for patients on critical care [10]. Besides, published recommendations from the International Society of Thrombosis and Hemostasis also suggests that hospitalized patients with COVID-19 pneumonia should receive pharmacological thromboprophylaxis with LMWH or unfractionated heparin (UFH) according to a risk stratification score and renal function, unless contraindicated.

Our case report suggests that COVID-19 patients treated with anticoagulants are at risk of developing spontaneous retroperitoneal hematoma. Although rare, it should remain as a possible source of bleeding, especially when patients present with flank pain, anemia and signs of hypovolemia. Close monitoring and early intervention may improve the outcome in this group of patients.

## References

[1] Malas MB, Naazie IN, Elsayed N, Mathlouthi A, Marmor R, Clary B. Thromboembolism risk of COVID-19 is high and associated with a higher risk of mortality: a systematic review and meta-analysis. EClinicalMedicine. 2020;29:100639. 33251499 10.1016/j.eclinm.2020.100639 PMC7679115

[2] Tang N, Bai H, Chen X, et al. Anticoagulant treatment is associated with decreased mortality in severe coronavirus disease 2019 patients with coagulopathy. J Thromb Haemost 2020;18:1094–9. 32220112 10.1111/jth.14817 PMC9906401

[3] Al-Samkari H, Karp Leaf RS, Dzik WH, Carlson JCT, Fogerty AE, Waheed A, et al. COVID-19 and coagulation: bleeding and thrombotic manifestations of SARS-CoV-2 infection. Blood. 2020;136:489–500. 32492712 10.1182/blood.2020006520 PMC7378457

[4] Ottewill C, Mulpeter R, Lee J, Shrestha G, O’Sullivan D, Subramaniam A, et al. Therapeutic anti-coagulation in COVID-19 and the potential enhanced risk of retroperitoneal hematoma. QJM 2021;1–3. 33742677 10.1093/qjmed/hcab059 PMC8083784

[5] Patel I, Akoluk A, Douedi S, Upadhyaya V, Mazahir U, Costanzo E, et al. Life-threatening psoas hematoma due to retroperitoneal hemorrhage in a COVID-19 patient on enoxaparin treated with arterial embolization: a case report. J Clin Med Res. 2020;12:458–61. 32655742 10.14740/jocmr4256 PMC7331864

[6] Conci S, Ruzzenente A, Donadello K, Cybulski AJ, Pedrazzani C, Campagnaro T. Haemodynamic instability in a critically ill patient with COVID-19 pneumonia: searching over the chest - report of a clinical case and mini-review of the literature. Case Rep Imag Surg. 2020;3:1–3.

[7] Nakamura H, Ouchi G, Miyagi K, Higure Y, Otsuki M, Nishiyama N, et al. Case report: iliopsoas hematoma during the clinical course of severe COVID-19 in two male patients. Am J Trop Med Hyg. 2021;104:1018–21. 10.4269/ajtmh.20-1507 PMC7941852 33534775

[8] Javid A, Kazemi R, Dehghani M, Bahrami SH. Catastrophic retroperitoneal hemorrhage in COVID-19 patients under anticoagulant prophylaxis. Urol Case Rep. 2021;36:101568. 33520659 10.1016/j.eucr.2021.101568 PMC7829136

[9] Chan YC, Morales JP, Reidy JF, Taylor PR. Management of spontaneous and iatrogenic retroperitoneal haemorrhage: conservative management, endovascular intervention or open surgery? Int J Clin Pract 2008;62:1604–13. 17949429 10.1111/j.1742-1241.2007.01494.x

[10] Gomez K, Laffan M, Bradburry C. Debate: should the dose or duration of anticoagulants for the prevention of venous thrombosis be increased in patients with COVID-19 while we are awaiting the results of clinical trials? Br J Haematol 2021;192:459–66. 33236402 10.1111/bjh.17241 PMC7753713

